# Mitochondrial p32 is upregulated in Myc expressing brain cancers and mediates glutamine addiction

**DOI:** 10.18632/oncotarget.2708

**Published:** 2014-12-22

**Authors:** Valentina Fogal, Ivan Babic, Ying Chao, Sandra Pastorino, Rajesh Mukthavaram, Pengfei Jiang, Yoon-Jae Cho, Sandeep C. Pingle, John R. Crawford, David E. Piccioni, Santosh Kesari

**Affiliations:** ^1^ Translational Neuro-oncology Laboratories, Moores Cancer Center, University of California San Diego, La Jolla, CA; ^2^ Division of Neuro-Oncology, Department of Neurosciences, University of California, San Diego, La Jolla, CA; ^3^ Stanford University, Palo Alto, CA

**Keywords:** mitochondrial p32, c-Myc, glutamine addiction, brain cancer

## Abstract

Metabolic reprogramming is a key feature of tumorigenesis that is controlled by oncogenes. Enhanced utilization of glucose and glutamine are the best-established hallmarks of tumor metabolism. The oncogene c-Myc is one of the major players responsible for this metabolic alteration. However, the molecular mechanisms involved in Myc-induced metabolic reprogramming are not well defined. Here we identify p32, a mitochondrial protein known to play a role in the expression of mitochondrial respiratory chain complexes, as a critical player in Myc-induced glutamine addiction. We show that p32 is a direct transcriptional target of Myc and that high level of Myc in malignant brain cancers correlates with high expression of p32. Attenuation of p32 expression reduced growth rate of glioma cells expressing Myc and impaired tumor formation *in vivo*. Loss of p32 in glutamine addicted glioma cells induced resistance to glutamine deprivation and imparted sensitivity to glucose withdrawal. Finally, we provide evidence that p32 expression contributes to Myc-induced glutamine addiction of cancer cells. Our findings suggest that Myc promotes the expression of p32, which is required to maintain sufficient respiratory capacity to sustain glutamine metabolism in Myc transformed cells.

## INTRODUCTION

It is increasingly clear that the genetic and molecular alterations associated with oncogenes and tumor suppressors are directly linked to aberrant metabolic activities of malignant compared to normal cells [1, 2]. Cancer cells frequently exhibit high glycolytic rates and avidly metabolize glucose to lactate in the presence of oxygen, a process classically referred to as the ‘Warburg effect’ or aerobic glycolysis [3]. Although this high rate of glycolysis was originally believed to derive from impaired mitochondrial metabolism, it is now clear that other aspects of mitochondrial function are essential for tumorigenesis and tumor progression, since elevated glycolysis alone is not sufficient for cancer cell survival [2, 4–11]. Mitochondrial metabolism includes activities of the tricarboxylic acid (TCA) cycle and the electron transport chain (ETC) of the oxidative phosphorylation (OXPHOS) process. In rapidly proliferating cells and cancer, the TCA cycle and OXPHOS provide both bioenergetic and biosynthetic activities for the production of macromolecule precursors (lipids, proteins, nucleic acids) needed to support cellular proliferation. In this regard, glutamine becomes an essential nutrient for cancer cells. Not only does glutamine provide nitrogen for protein and nucleotide synthesis, but, once catabolized to the TCA intermediate α-ketoglutarate (α-KG), it allows tumor cells to sustain TCA cycle activity, referred to as ‘anaplerosis’ [12].

Enhanced glucose [13], glutamine [14, 15] and lipid metabolism [16, 17] is a hallmark of malignant brain tumors. Grade IV astrocytic tumors (glioblastoma, GBM) are considered essentially incurable and among the most lethal human malignancies [18]. Together with high grade gliomas, medulloblastomas, neuroepithelial malignant tumors of the cerebellum, are the most frequent solid tumors in children and the leading cause of childhood cancer death. As such, elucidating the pathogenesis of brain tumors is critical for the development of effective therapies. Targeting glioma metabolic dependencies could be a novel and promising therapeutic strategy to treat these cancers. Compared to glucose metabolism, the signaling pathways that regulate glutamine metabolism in brain tumors are relatively uncharacterized [19, 20].

Deregulation of c-Myc (hereafter, ‘Myc’) transcription factor is closely correlated with the grade of brain tumor malignancy [21–28]. Oncogenic Myc regulates genes involved in glycolysis [29, 30], mitochondrial biogenesis and function [31], and glutamine metabolism [32, 33]. Myc not only diverts glucose-derived pyruvate from mitochondrial metabolism to lactate via stimulation of glycolysis, but it also maintains mitochondrial TCA cycle activity and cell viability by triggering a switch that utilizes glutamine as the oxidizable substrate within mitochondria. Pharmacological inhibition of glutaminase, the enzyme that catalyzes the first reaction of glutamine anaplerosis, can impair the growth of tumor xenografts from Myc-expressing B cells [34–36]. These findings indicate that reprogrammed glutamine metabolism is critical for the growth and survival of Myc-driven malignancies. While upregulation of glutamine surface transporters and glutaminase have been implicated in Myc-mediated regulation of glutamine metabolism [32, 33], there are undoubtedly additional players that facilitate enhanced glutamine oxidation.

The p32 protein (also known as complement component 1, q subcomponent binding protein – C1QBP) sustains mitochondrial OXPHOS by playing a role in the synthesis of a number of mitochondrial-encoded electron transport complex subunits [37–39]. Although the primary subcellular localization of p32 is the mitochondrial matrix, it is also found at the cell surface (a localization that appears to be tumor-specific [40]). Many tumors exhibit higher expression levels of p32 than their non-malignant tissues [40–42] suggesting a potential role for p32 in tumorigenesis. Loss of p32 function decreased both cellular respiration and tumorigenesis [37], indicating that p32 mitochondrial function is essential for tumor formation. Intriguingly, genome wide analysis using microarrays, serial analysis of gene expression (SAGE) and chromatin immunoprecipitation (ChIP) studies have indicated p32 among the list of genes that are transcriptionally regulated by Myc [43–46].

Here, we describe a role of the p32 protein in the stimulation of glutamine metabolism by Myc in brain tumors. We demonstrate that p32 is a direct transcriptional target of Myc and that its expression contributes to Myc-induced glutamine addiction of cancer cells. We show that p32 levels are increased in a variety of brain cancers and that they closely correlate with the malignancy grade and Myc expression levels. Attenuation of p32 expression in glioma cell lines and in patient-derived human glioma cells impairs cell growth *in vitro* and tumor development *in vivo*. Taken together these data provide more mechanistic insight into the reprogramming of glutamine metabolism, and how this is sustained in the pathogenesis of brain tumors.

## RESULTS

### P32 is over-expressed in malignant brain cancers and correlates with Myc expression

**Figure 1:**
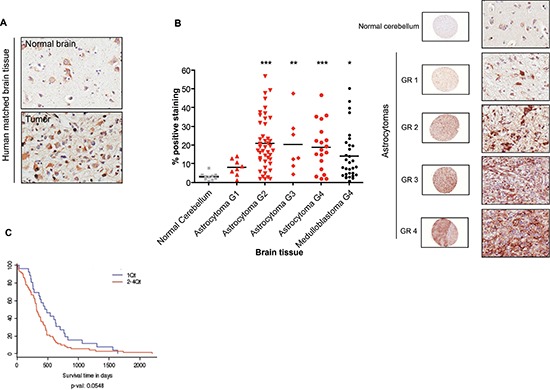
Upregulation of p32 in malignant brain tumors Matched normal and malignant brain tissue **(A)**, and a human brain tumor array **(B)** were stained with a polyclonal anti-p32 antibody. P32 positive staining for each core was quantified using Aperio software **(B left panel)**. The array contains over 100 cases of gliomas, including both astrocytoma and medulloblastoma subtypes, and normal tissue. The two-tailed Student t test was used for statistical analysis. Significant differences are indicated using the standard Michelin Guide scale (*p* < 0.05 (*), significant; *p* < 0.01 (**), highly significant; *p* < 0.001 (***) extremely significant. Representative cores, at low (10x) and high (400x) magnification, of normal cerebellum and astrocytomas of different malignancy grade (GR) are indicated on the right panel of Figure 1B. **(C)** Increasing expression of p32/C1QBP correlates with poor survival. Shown is a survival plot for mesenchymal subtype of GBM based on increasing expression of p32/C1QBP using Affymetrix HT_HG-U133A. Plot generated using the Glioblastoma Bio Discovery Portal (http://robtcga.nci.nih.gov/#genes).

Immunohistochemical analysis of matched normal and malignant brain tumor tissues from patients revealed higher p32 reactivity in tumor tissue compared to normal brain (Fig. 1A upper panels). Staining was predominantly cytoplasmic, but membrane staining was also evident. In normal brain tissues, p32 expression was confined to pyramidal neurons. In agreement with these observations, a survey of a human brain tumor tissue array revealed that p32 is upregulated in most glioma cases, with significant enhanced expression in malignant (grade 2, 3 and 4) compared to both normal and hyperplastic (G1) tissues (Fig. 1B). To assess clinical relevance of increased expression of p32 in malignant brain tumors, we examined correlation between patient survival and p32 expression level. Analysis of TCGA data using the GBM Bio Discovery Portal (http://robtcga.nci.nih.gov/#genes) revealed a significant correlation between high p32 expression and decreased survival (*p* = 0.0548) (Fig. 1C).

**Figure 2:**
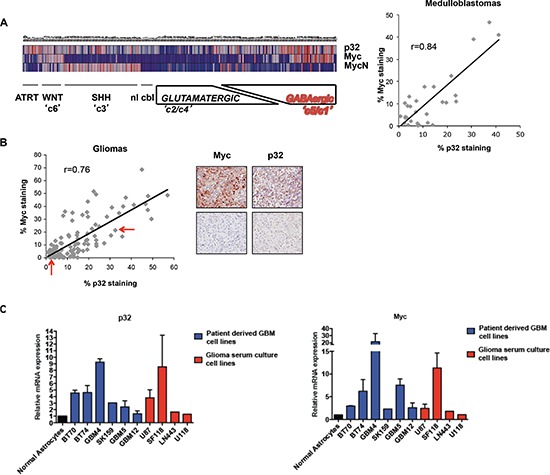
Correlation between p32 and Myc expression in human gliomas and glioma cell lines **(A)** Correlation between Myc and p32 expression in medulloblastoma human tumors. Left panel. Heat map of p32, c-Myc and N-Myc for each medulloblastoma subgroup (c1 through c6) and an additional ATRT subgroup (atypical teratoid/rhabdoid tumors) [23], C5/c1 Medulloblastoma subgroup, characterized by a Myc activation signature [23], exhibits high expression of p32. WNT, Wingless signaling pathway, SHH Sonic Hedgehog signaling pathway. nl cbl, Normal cerebellum samples. **(A)**-right panel and **(B)**: correlation between p32 and Myc expression in a medulloblastoma array **((A)**-right panel) and a mixed glioma **(B)** as indicated by the % of p32 and Myc positive staining for each core of the arrays. Sequential slides of each array were stained separately with polyclonal anti p32 and c-Myc antibodies. The % of p32 and Myc positive staining for each core was quantified using Aperio software. The Pearson correlation coefficient (r) of linear regression was calculated using data sets deprived of samples expressing low Myc (<15% of staining) but moderate-high p32 (>15% of staining). The immunohistochemistry images (200x magnification) show representative glioma cores (red arrows in graph) exhibiting correlation between Myc and p32 expression. **(C)** qPCR analysis of p32 and Myc expression in established glioma cell lines (red) and patient derived glioma stem cells (blue) as compared to normal astrocytes (black). The bars shown are normalized to an internal β-actin control and represent the mean ± SEM of at least three independent experiments.

Myc is central to the genesis of most human cancers, and deregulated Myc is closely correlated with the grade of brain tumor malignancy [21–23, 47]. Microarray analysis of Myc-responsive genes identified p32 as a potential transcriptional target of Myc [43, 44, 46]; as such, we investigated a possible correlation between Myc and p32 expression in malignant brain tumors. We first focused on medulloblastoma. These highly heterogeneous malignant brain tumors, usually found only in children, have been classified into six molecular subgroups, each with a unique combination of chromosomal aberrations [23]. One molecular subgroup, with a particularly aggressive course, is characterized genetically by MYC copy number gains and transcriptionally by enrichment of photoreceptor pathways. Unsupervised clustering of mRNA expression data from 194 medulloblastoma revealed concomitant high expression of p32 and Myc in medulloblastoma with poor clinical outcome (Fig. 2A left panel-c5/c1 subgroup). Correlation of p32 and Myc expression in medulloblastoma tissues was also evident following immunostaining of a medulloblastoma tissue array (Fig. 2A right panel). Similar immunohistochemical analysis was also performed in an array containing glioma subtypes (Fig. 2B). In this case the correlation had a lower Pearson coefficient (*r* = 0.49) because some tissue cores express low or undetectable levels of Myc, but moderate to high levels of p32 (Supplementary Fig. S1, samples in red box). This is not surprising, since p32 expression is also likely to be regulated by Myc-independent mechanisms. A linear regression analysis excluding these tissues revealed a strong correlation between Myc and p32 expression (*r* = 0.76) (Fig. 2B). In addition, quantitative RT-PCR analysis showed an upregulation of p32 in glioma cell lines (Fig. 2C red bars) as well as patient-derived glioma stem cells (Fig. 2C, blue bars) compared to normal astrocytes. In agreement with results from the tissue arrays, there was a strong correlation between up-regulation of Myc and p32 in over half of the cell lines tested.

### P32 is transcriptionally upregulated by Myc

**Figure 3:**
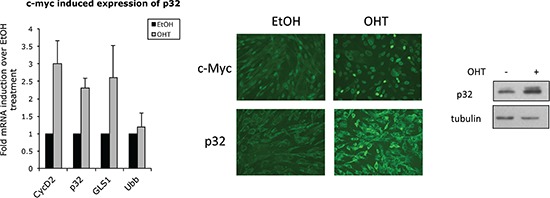
Myc promotes p32 expression Left panel: qPCR analysis of target genes from total RNA isolated from MRC5 MycER cells treated with 250 nM OHT or vehicle (EtOH) for 24 hrs. Ubiquitin (Ubb) is included as a negative control while cyclin D2 (CycD2) and glutaminase (GLS1) are positive controls. The bars shown are normalized to an internal β-actin control and represent the mean ± SD of at least three independent experiments. Fold of each gene induction over control treatment is indicated. Middle panel: immunofluorescence staining of Myc and p32 24 hrs post-OHT treatment. The immunoblot on the right shows upregulation of p32 protein upon Myc induction. Tubulin was used as loading control.

Genome-wide analysis using microarrays and serial analysis of gene expression (SAGE) [43, 46], and, more recently, a combination of expression profiling and ChIP-chip analysis [44] identified p32 (C1QBP) as one of the Myc target genes. Collectively these studies, together with our correlation data (Fig. 2), suggest that Myc directly affects p32 expression. To study the effect of Myc activation on p32 transcription and protein level, we used immortalized MRC5 cells stably expressing Myc fused to the oestrogen receptor ligand-binding domain (MycER). A 24-h treatment with 4-hydroxy tamoxifen (OHT) lead to a significant upregulation of *p32* that was comparable to those observed for the established Myc targets, *Cyclin D2* and *glutaminase 1* (Fig. 3, left panel). Accordingly, p32 protein levels were also increased upon Myc induction, as indicated by immunofluorescence staining and immunoblot (Fig. 3). Considering that the MycER system is to some extent “leaky” (see some basal nuclear localization in Myc staining of Fig. 3 middle panel) it is possible that the true fold increase of p32 expression following Myc activation is higher than that reported by the system.

**Figure 4:**
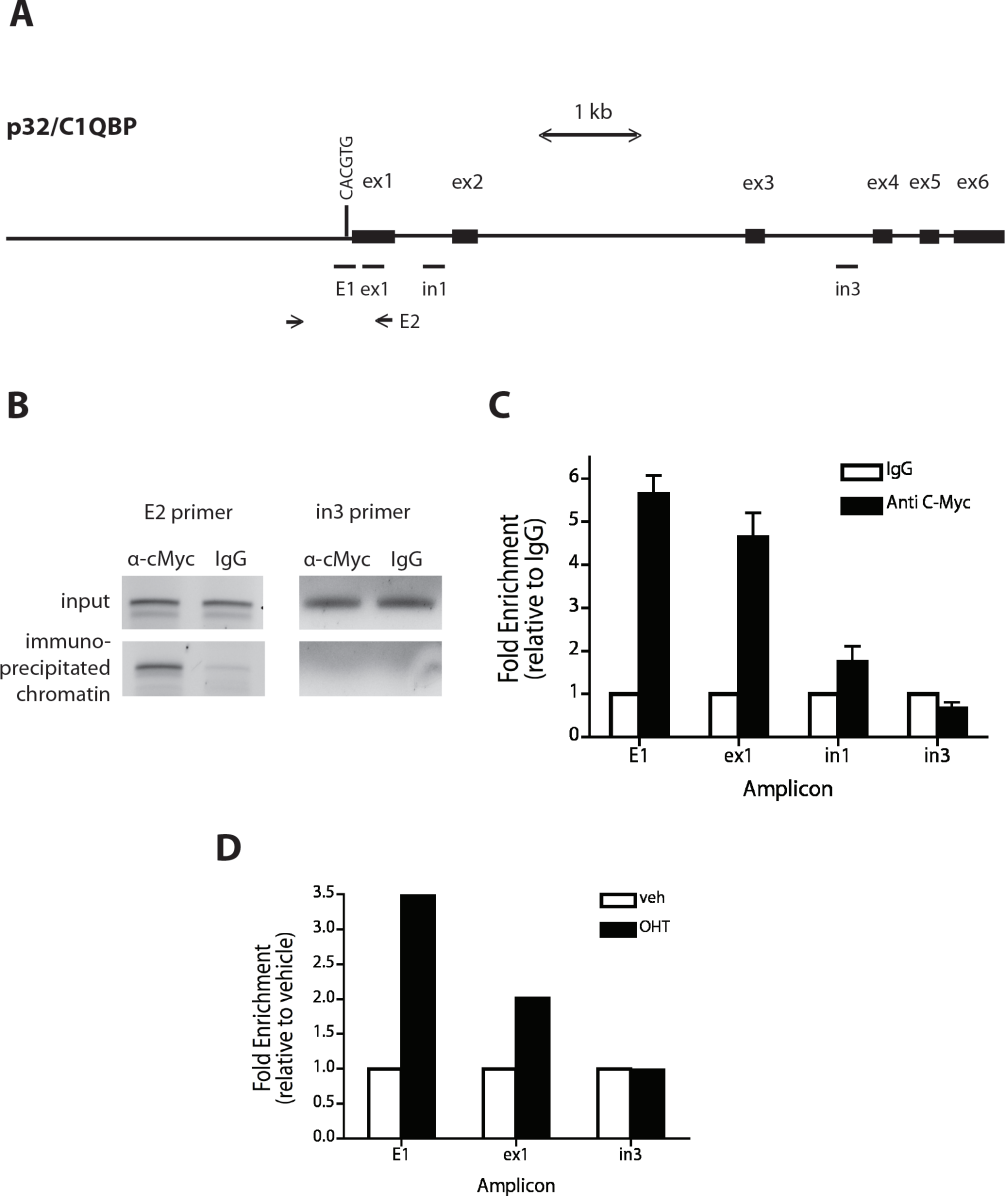
Myc binds to the *p32* promoter **(A)** Schematic of the human *p32* promoter sequence starting from 3 kb upstream of exon 1 to exon 6 of the *c1qbp* gene. Exons (ex) are represented by black boxes and the E box is indicated with a vertical bar. Horizontal bars indicate the regions amplified for scanning ChIP analysis. E2 is the *p32* promoter region containing the E-box and amplified by conventional PCR (Figure 3B). **(B and C)** A ChIP assay was performed on SF188 cells with anti Myc antibody and IgG as a control. Precipitated chromatin was PCR-amplified using E2 primers **(B)**. Quantitative PCRs were performed to amplify and quantify E1, ex1, in1, and in3 promoter regions. Shown are averages with standard deviations of triplicate independent experiments. Binding to amplicons is shown as a percentage of total input DNA plotted relative to the signal obtained from IgG precipitation. **(D)** Quantification of MycER binding to the p32 promoter after addition of OHT. MRC5 MycER cells were serum starved for 24 hrs and either treated with vehicle (EtOH) or 250nM OHT for 4 hours. Subsequently ChIP assays were conducted as described in (B). The bar graph presented is indicative of three independent experiments.

Myc is known to bind to a canonical consensus DNA sequence CACGTG, termed the E-box, but can also bind several other non-canonical DNA motifs [48]. Analysis of the p32 promoter sequence identified several described consensus sequences for Myc binding (not shown), with an E-box among them, just upstream (−24 to −19 bp) of the p32 transcriptional start codon (Fig. 4A). We used ChIP to test whether p32/C1QBP may be a direct downstream target of Myc transactivation. Using SF188 cells and primers flanking the E-box, we found that the *p32* promoter was significantly enriched in the Myc ChIP sample compared to IgG control (Fig. 4B). Primers amplifying other p32 promoter regions (ex1, in1 and in3 Fig. 4B and 4C) identified specific Myc binding in regions proximal but not distal to the E-box. To further confirm these data, a ChIP analysis was performed in MRC5 MycER cells. Compared with vehicle-treated cells, activation of MycER by OHT led to a 3.5-fold enrichment in p32 promoter binding at the E-box (Fig. 4D). There was no binding enrichment in an area of p32 gene distal to the E-box. Taken together, these data indicate that Myc protein can directly bind to a regulatory region of p32 promoter suggesting that this gene is a direct target of Myc.

### P32 stable knockdown impairs glioma cell proliferation

**Figure 5:**
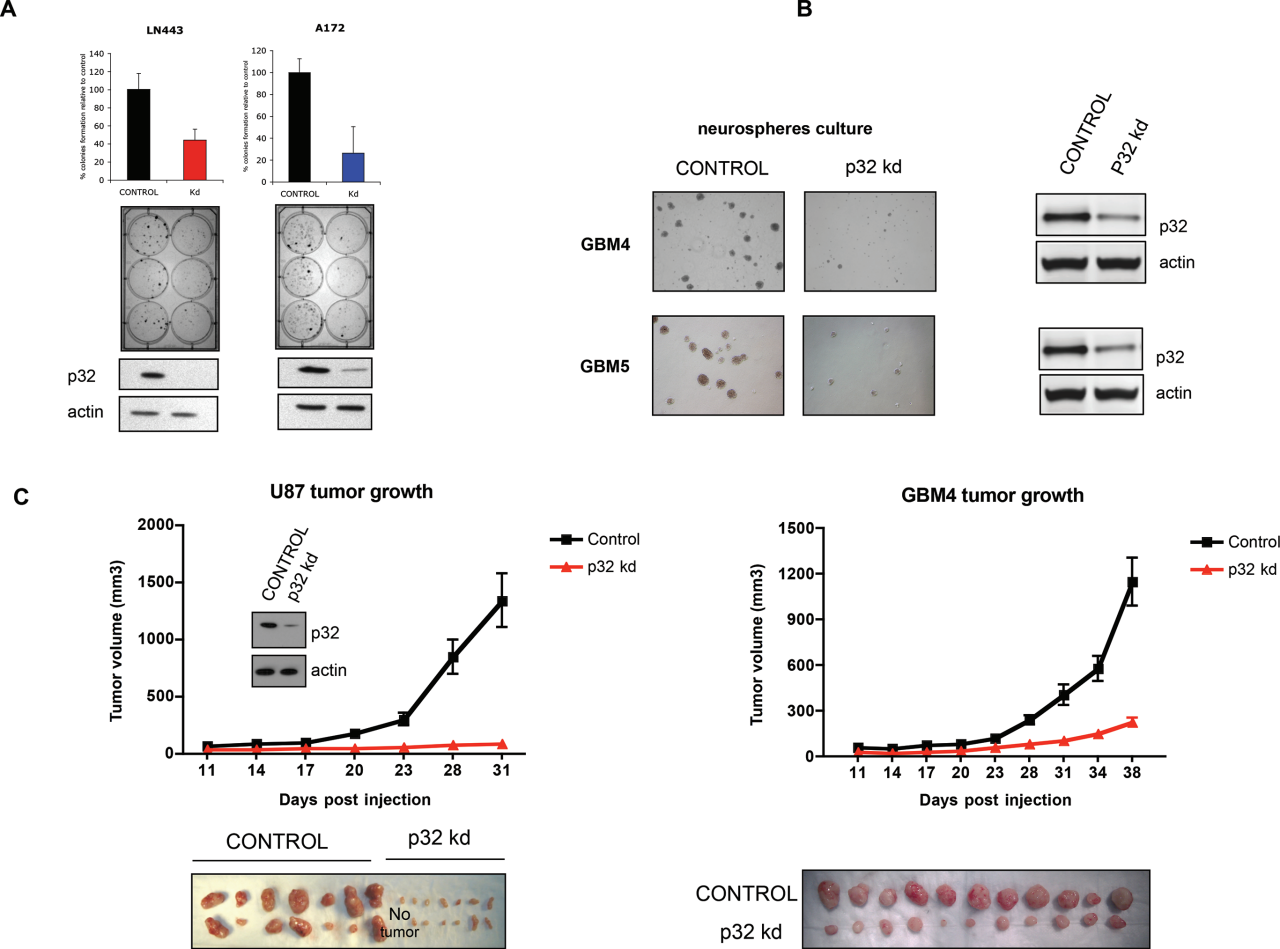
Attenuation of p32 expression reduces glioma cell proliferation and tumor growth **(A)** Upper panels: colony formation assay of control and p32 knockdown stable cell lines. The graphs represent the mean ± SD of at least three independent colony formation assays each performed in triplicate (middle panel). Lower: Western blot analysis demonstrating p32 stable knockdown in the indicated glioma cell lines. **(B)** Microscopic analysis of p32 knockdown and control GBM4 and GBM5 glioma stem cell-like neuropheres. Right panel-western blot showing efficient p32 knockdown in the indicated glioma stem cell-like cells. **(C)** Tumor growth properties of U87 cells (left panel) and GBM4 glioma stem cell-like cells (right panel) infected with viruses encoding control or p32 kd shRNA. The graphs represent the mean ± SEM (*p* < 0.001) of tumor volumes as a function of time. The lower panels show pictures of tumors from each group (*n* = 14 or *n* = 11) at the end point.

To assess the impact of p32 on glioma cell proliferation, we generated stable glioma cell lines with attenuated p32 expression (Fig. 5A lower panels). The p32 knockdown (p32 kd) cell lines exhibit reduced proliferation rate as revealed by colony formation assays (Fig. 5A upper panels). Furthermore, stable transduction of patient-derived glioma stem cell-like lines with p32 shRNA lentivirus significantly reduced neurosphere formation when compared to control shRNA infected cells (Fig. 5B). Significantly, p32 kd glioma cells and patient derived cell lines produced smaller tumors *in vivo* compared to control (Fig. 5C).

### P32 plays a role in Myc-induced glutamine addiction of cancer cells

**Figure 6:**
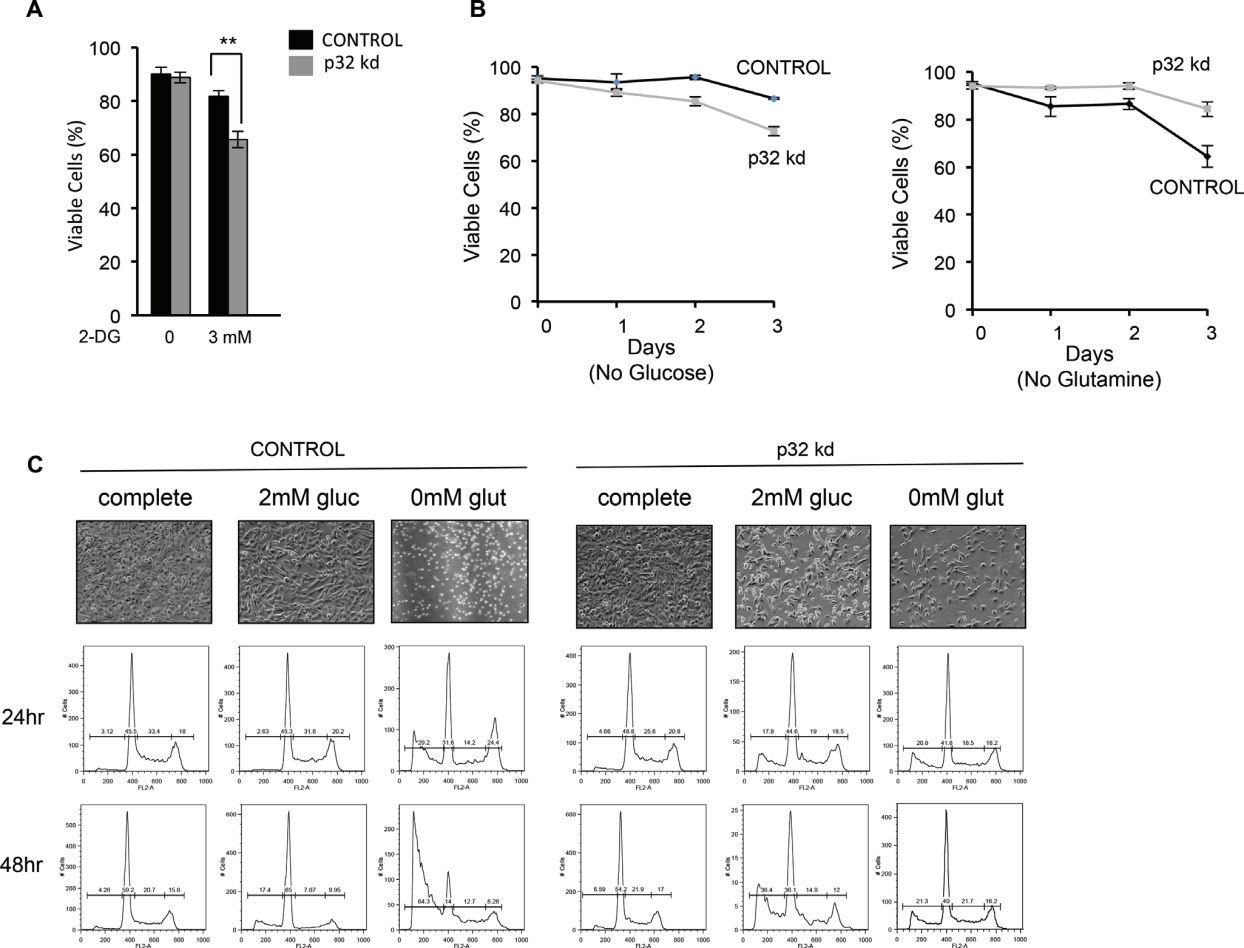
Loss of p32 sensitizes cells to glucose withdrawal and reduces sensitivity to glutamine deprivation **(A)** p32 knockdown cells are more sensitive to the glycolytic inhibitor 2-DG. SF188 p32 knockdown cells were plated in low glucose media (2.5 mM) and 3mM 2-DG. After 18 h cell viability was determined by trypan blue exclusion. Data is the average of three experiments ± SD. **(B)** SF188 Control or p32 knockdown cells were plated in glutamine or glucose free media. Cell viability was determined by trypan blue exclusion. Each time point is the mean of three experiments ± SD. **(C)** Control (left) and p32 knockdown (right) SF188 cells were plated in complete media (25mM glucose and 4mM glutamine) and subsequently cultured in either complete media or media with 2mM glucose or without glutamine. Cell viability was determined at the indicated time points by FACS analysis of PI stained cells. Upper panel-Microscopic analysis of p32 knockdown and control SF188 cells after 2 days of growth in the indicated tissue culture media. The shown result is representative of three independent experiments.

Loss of p32 in breast cancer cells has been shown to promote a shift from OXPHOS toward aerobic glycolysis [37]. We examined the metabolic effects of p32 knockdown on SF188 glioblastoma cells. Attenuation of p32 expression in glioma cells resulted in enhanced glucose consumption and lactate production, two hallmarks of aerobic glycolysis (Fig. S2, supplementary data). Dependence of p32 knockdown cells on glycolysis was confirmed by an enhanced sensitivity to the non-metabolizable glucose analogue, 2-deoxyglucose (2DG) (Fig. 6A). Furthermore, p32 loss reduced growth and viability of glioma cells in glucose free and low glucose media respectively (Fig. 6B left panel and Fig. 6C).

**Figure 7:**
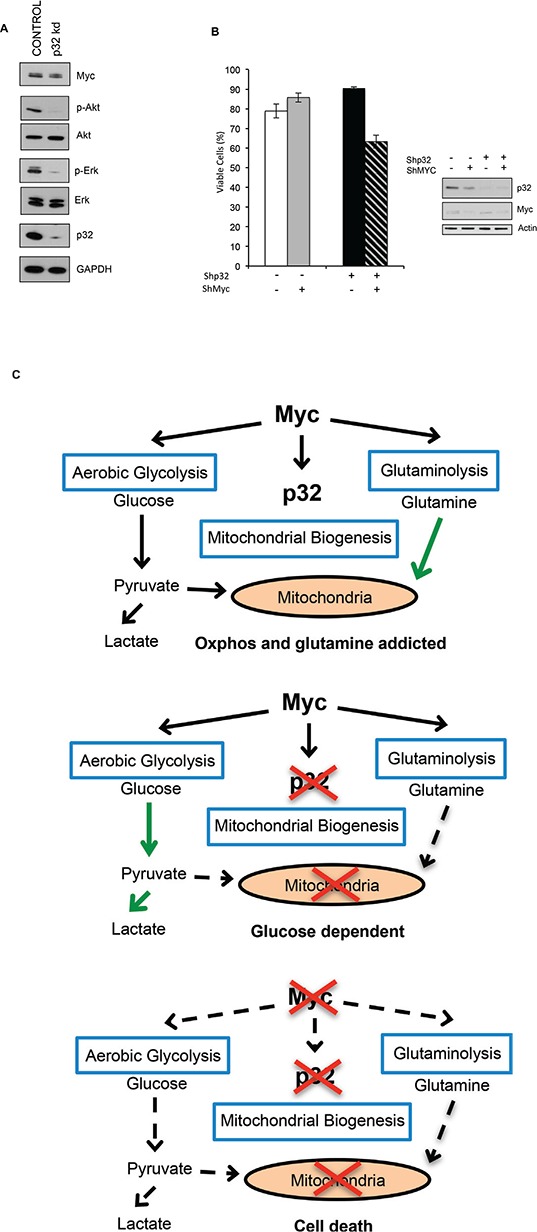
P32 is required for Myc-dependent glutamine metabolism **(A)** Immunoblot detecting expression of p32, Myc, Erk, phospho Erk (p-Erk), Akt, and phospho-Akt (p-Akt) in lysates from Control and p32 knockdown SF188 cells. **(B)** Myc was knocked down in both SF188 ShRNA Control and ShRNA p32 cells. Cells were then plated in glutamine free media and 24 hrs later cell viability was determined by trypan blue exclusion. Data shown is the mean of three experiments ± SD. **(C)** Model for p32 role in cancer cell metabolism downstream of Myc. Myc promotes the expression of p32, which helps maintain OXPHOS and glutamine addiction. Loss of p32 results in a dependence on glucose. Loss of both Myc and p32 inhibits cell growth.

SF188 cells are derived from a brain tumor characterized by Myc amplification [49]. These cells exhibit a glutaminolytic phenotype that correlates with Myc-dependent cellular addiction to glutamine metabolism for survival [33]. Myc inhibition in these cells is associated with reduced sensitivity to glutamine withdrawal [33]. We examined if loss of p32 impacts Myc-dependent glutamine metabolism. SF188 cells depleted of p32 exhibited enhanced uptake of glutamine (Fig. S2, supplementary data). Interestingly, p32 knockdown in these Myc-induced glutamine addicted cells displayed reduced sensitivity to glutamine withdrawal (Fig. 6B right panel and 6C). However, resistance to glutamine deprivation in p32 knockdown cells was not due to changes in Myc expression levels, as western blot analysis of SF188 cell lysates revealed equal amounts of Myc protein in control versus p32 knockdown lysates (Fig. 7A). We examined if enhanced glucose metabolism and sensitivity to glucose withdrawal in p32 knockdown cells was a consequence of activated PI3K/Akt [50] or ras/MAPK signaling [51]. Consistent with results reported for lung adenocarcinoma cell lines [52], p32 knockdown glioma cells showed significant decrease in Akt and Erk phosphorylation (Fig. 6A). Thus the increase in glucose uptake and metabolism was not a result of altered oncogenic signaling.

Given that Myc stimulates both glucose and glutamine metabolism, we hypothesized that loss of p32 in Myc-addicted cells switches metabolism from glutamine-addiction to glucose-addiction. To test this hypothesis, SF188 control and p32 knockdown cells were transduced with lentivirus containing a Myc-ShRNA (shMyc) or a GFP shRNA (shCTRL) and the cells were incubated in glutamine-depleted medium. In agreement with previously published data [33], control SF188 cells transduced with Myc-shRNA exhibited increased resistance to glutamine starvation compared to cells transduced with control-ShRNA (Fig. 7B). However, when Myc was knocked down in cells lacking p32, the enhanced resistance to glutamine withdrawal was lost. These findings suggest that growth of p32 depleted cells on glucose as the sole carbon source requires Myc.

## DISCUSSION

Mitochondrial metabolism is reprogrammed by oncogenes to support macromolecular synthesis and anabolic growth [2, 53]. Essential for anabolic growth is the amino acid glutamine whose utilization through the mitochondria is induced by Myc oncogene [32, 33]. Myc diverts glucose-derived pyruvate away from mitochondrial metabolism and enhances cellular dependence on glutamine that is necessary for fatty acid synthesis and TCA cycle anaplerosis [12]. Myc transformed cells are thus highly vulnerable to alterations in glutamine metabolism. Although glutamine analogues have shown clinical activity, their use was discontinued due to significant neurotoxicity [54]. This suggests that selective inhibition of critical players of glutamine metabolism might reproduce the anti-cancer effects and avoids the non-specific toxicity of general inhibition of glutamine metabolism. As such, understanding the mechanism by which Myc transforms cells to an addiction to glutamine can identify potential targets to inhibit the growth of glutamine addicted cancers. Here we report that p32/C1QBP is upregulated by Myc in malignant brain tumors. Importantly, we demonstrate that loss of mitochondrial-localized p32 overcomes Myc-induced glutamine addiction (Fig. 7). Additionally, loss of p32 sensitizes cells to glucose deprivation and glycolytic inhibitors (Fig. 6).

We demonstrate that p32 is upregulated in adult and pediatric brain cancers (glioblastoma and medulloblastoma) and its upregulation correlates significantly with Myc expression (Fig. 1 and 2). Genome-wide analyses using microarrays and SAGE [43, 46], and, more recently, a combination of expression profiling and ChIP-chip analysis [44], have indicated p32 among Myc targets. We now demonstrate that Myc directly associates with the promoter of p32 (Fig. 4) and regulates p32 transcript levels (Fig. 3). Collectively these studies suggest that Myc is a direct transcriptional activator of p32 expression. ShRNA-mediated stable knockdown of p32 in glioma cell lines and patient-derived tumor initiating cells impaired cell proliferation *in vitro* and tumorigenic potential *in vivo* in xenograft mouse models. These data are consistent with previous reports for other cancers such as lung and breast cancers [37, 52]. We further demonstrate p32 to play a role in mediating Myc–induced glutamine metabolism. Attenuation of p32 expression reduced glioma cell sensitivity to glutamine deprivation (Fig. 6 and 7), but induced a glucose dependent phenotype (Fig. 6 and [37]). Together, the data suggest that p32 acts downstream of Myc and modulates glutamine metabolism (Fig. 7C). Myc upregulation of p32 is part of the metabolic reprogramming towards glutaminolysis. Therapeutic targeting of p32 therefore may be a relevant strategy to switch tumors to a glucose-dependent phenotype and ultimately improve the efficacy of treatments that interfere with tumor glucose metabolism.

Myc has been linked to increased rates of mitochondrial biogenesis primarily via activation of target genes encoding master regulators of mitochondrial function, biosynthesis and metabolism such as NRF1, PGC-1β and TFAM [31, 44, 55, 56]. Both mitochondrial biogenesis and oxidative phosphorylation require not only increased expression of nuclear-encoded proteins that are imported into the mitochondria, but also the transcription of the 13 polypeptides, constituents of the ETC, encoded by the mitochondrial genome. Myc itself does not appear to function as a transcriptional regulator of the mitochondrial genome; however, it directly binds and increases the expression of the critical mitochondrial transcription factor TFAM [31]. P32 expression likely provides a tumor proliferative advantage in Myc expressing cells through mitochondrial biogenesis and/or maintaining mitochondrial integrity. P32 has been reported to regulate the translation of mitochondrial-encoded genes [37, 39]. Additionally, p32 function in maintaining OXPHOS may be required to regenerate NAD+ necessary for continued glutaminolysis [12].

Glutamine is well established as a key substrate that supports energy production and biosynthetic reactions in cancer cells [57]. Myc control of glutamine metabolism is exerted via multiple mechanisms. Myc activates the transcription of high-affinity glutamine transporters (ASCT2 and SN2) and also upregulates the expression of mitochondrial glutaminase (GLS) [32, 33, 35]. Related to Myc control of glutamine metabolism is Myc-dependent increased rate of mitochondrial biogenesis and function [31, 44]. Our data suggests that Myc-induced p32 expression may be important for metabolic reprogramming towards glutaminolysis in brain tumors. Indeed, Myc-induced addiction to glutamine as an energy source relies on efficient oxidation of glutamine within the mitochondria via the TCA cycle. Enhanced entry and flux of glutamine through the TCA cycle requires the continual regeneration of mitochondrial NAD+ through the activities of the mitochondrial ETC. Chemotherapeutic strategies using glycolytic inhibitors have been unsuccessful in arresting tumor proliferation and evidence has indicated that, although tumor cells maintain a high glycolytic rate, most tumor mitochondria are not defective in their ability to carry out oxidative phosphorylation. Instead, mitochondria metabolism is reprogrammed by oncogenes to support macromolecular synthesis and therefore anabolic growth [2]. Myc has been shown to activate the expression of genes involved in glycolysis and glutaminolysis but also in mitochondrial biogenesis and function [58]. Targeting p32 may be a relevant therapeutic strategy to inhibit mitochondrial metabolism by inhibiting the expression and function of the mitochondrial ETC, as is the case with metformin. In fact, antibody and nanoparticle targeting of p32 have proved successful at reducing tumor size validating it as an important therapeutic target [40].

## METHODS

### Reagents, cell lines and tumors

Polyclonal anti N-term p32 peptide (aa 76–93) antibody has been previously described (37). The following antibodies were from Cell Signaling Technology: Rabbit mAb c-Myc (D84C12) XP^TM^, Rabbit mAb p44/42 MAPK (Erk1/2), Rabbit mAb Akt, Rabbit mAb phospho-p44/42 MAPK (Erk1/2) (Thr202/Tyr204) (20G11), Rabbit mAb Phospho-Akt (Ser473), Rabbit mAb beta-actin (13E5), and Rabbit mAb GAPDH (DIGH11) XP^TM^. Tissue arrays of paraformaldehyde fixed, paraffin embedded tumor and normal tissue samples (multibrain cancer and normal adjacent tissue array GL1001, brain glioblastoma and normal tissue array GL806a, Medulloblastoma brain tissue array BC17012) were from US Biomax, Inc.

U87-MG, SF188, LN443, U118 and A172 are established human glioblastoma cell lines and were cultured in DMEM supplemented with 10% dialyzed fetal bovine serum (dFBS) (Hyclone), 1% glutamine pen-strep (Omega Scientific) at 37^o^C/5% CO_2_. GBM4, GBM5, GBM12, BT70 and BT74 are patient derived glioma stem cells and were obtained and cultured as neurospheres as previously described [59, 60]. GBM surgical samples were dissociated in stem cell isolation medium containing human recombinant EGF (20 ng/ul), human bFGF (10 ng/ul) and heparin (2 ug/ml), washed, filtered through a 30μm mesh and plated onto ultra-low adherence flasks at a concentration of 5×10^5^ to 1.5×10^6^ viable cells/ml. Sphere cultures were passaged by dissociation, washing and resuspension in neural stem cell culture medium (NeuroCult^TM^ NS-A Proliferation kit #05751, Stemcell Technologies), according to the manufacturer's instructions.

MRC5 immortalized human fibroblasts stably transfected with MycER (a kind gift of Dr. Gerald Evans University of California San Francisco) were cultured in DMEM supplemented with 10% dFBS, 25 mM glucose, 1% glutamine, and penicillin-streptomycin.

Lentivirus p32 and control shRNA constructs have been previously described [37]. The Myc ShRNA construct (shMyc F86) was a kind gift of Dr. Ralph DeBerardinis.

P32 deficient cell lines were obtained as previously described [37].

### Immunohistochemistry

Paraffin-embedded tissue array sections were deparaffinized and treated with antigen unmasking solution (Vector H-3300) according to the manufacturer's instructions. Peroxidase block was performed by incubating the slides for 20–30 min in 0.3% H_2_O_2_. After a rinse with H_2_O, slides were first blocked with diluted normal horse serum (R.T.U. Vectastain Elite ABC kit universal) for 20 min and subsequently with the Biotin/Avidin blocking system by Vector. Sections were then stained with the primary antibody (rabbit anti N-term p32 5μg/ml, rabbit anti Myc (1:200) in normal horse serum for 1 h at RT or overnight at 4°C. Antibody binding was detected after a 30-min incubation with biotinylated “universal” secondary antibody and VECTASTAIN RT Elite ABC reagent. Peroxidase substrate solution (DAB substrate kit) was incubated until desired stain intensity developed. Slides were finally counterstained with Hematoxylin (Vector H-3401). The slides were scanned on a Scanscope CM-1 scanner and subsequently processed for staining quantification using ImageScope software (Aperio Technology).

### Quantitative real time PCR (qPCR)

RNA was isolated from cells using the RNeasy mini kit from Qiagen. cDNA was synthesized from 800 ng RNA using Biorad iScript cDNA Synthesis kit. Quantitative real time PCR was performed using the Biorad CFX96 Real Time PCR detection system. Relative gene expression was normalized to actin. The primers used for qPCR are listed in Supplementary Table S1.

### Chromatin immunoprecipitation (ChIP) assay

Chromatin immunoprecipitation was performed with SF188 and MRC5 MycER cells using the SimpleChIP(R) Enzymatic Chromatin IP Kit (Cell Signaling Technology, Cat. No. 9002) according to the manufacturer's instructions. Cell lysates were incubated with anti c-Myc antibody (Cell Signaling Technology) or control Rabbit IgG (Vector Lab). Precipitated DNA fragments were quantified using the primers listed in Supplementary Table S2.

### Colony formation assay

5000 cells were seeded in 6 wells plates and monitored for small colonies formation. After 2–3 weeks, cells were washed in PBS, fixed in 3.7% formaldehyde for 15 min at RT and subsequently stained for 30min-1 h with a 0.2% (w/v) solution of crystal violet in 20% methanol/H_2_O. Excess of dye was removed by extensive washes in H_2_O.

### Cell viability

SF188 control or p32 knockdown cells were plated at 5×10^4^ cells per well in a 12 well plate in glucose or glutamine free media in the absence of sodium pyruvate with 1% dFBS. Cell viability was determined at different time points by trypan blue exclusion. For the glycolytic inhibitor 2-DG experiments 5×10^4^ cells were grown in low glucose (2.5 mM) media with 1% FBS with 3 mM 2-DG. After 18 hours cell viability was determined by trypan blue exclusion.

For quantification of cell death by FACS analysis, 5×10^5^ cells per well were seeded in 6 wells plates in DMEM (25mM glucose-4mM glutamine)-10% dialyzed FBS and allowed to adhere overnight. The medium was removed by washing and replaced with glucose or glutamine free DMEM supplemented with 10% dFBS and either 25mM glucose/4mM glutamine, or 2mM glucose/4mM glutamine, or 25mM glucose/0mM glutamine. After 12, 24 and 48 hrs media containing floating cells was collected and combined to remaining cells detached by trypsinization. Cells were washed twice by centrifugation (200xg, 5 Min 4°C) in PBS and subsequently were resuspended at 2×10^6^ cells/1 ml of ice cold PBS in silanized polypropylene tubes to minimize sticking. 9ml of a −20°C cold solution of 70% ethanol was then added slowly by vortexing. After incubation ON at −20°C, cells were centrifuged at 200xg, 10 min 4°C and washed once with cold PBS. For Propidium iodide (PI) staining cells were resuspended in 500μl of PI/triton X-100 freshly prepared staining solution (0.1% Triton X-100 (Sigma) in PBS, 0.2mg/ml DNAse free RNAse A (Sigma) and 20μg/ml PI (Roche)) and incubated at 37°C for 15 min. Data was acquired via flow cytometry (Canto, BD FACS), and the percentage of cells in G_0_/G_1_, S and G_2_ phases were calculated by ModFit LT software version 3.0.

### Mice and tumors

To produce tumors, 2 ×10^6^ cells in 100 μl of PBS were injected subcutaneously into the flank of NSG mice (Jackson Laboratory). Tumor volume was calculated using the equation volume (mm3) = **d**2 × **D**/2, where **d** and **D** are the smallest and biggest tumor diameters, respectively. All animal experimentation received approval from the Animal Research Committee from UCSD.

### Statistical analysis

Data are expressed as means ± standard deviations (SD) for *in vitro* experiments and as means ± standard errors of the means (SEM) for *in vivo* experiments. The two-tailed Student t test was used for all statistical analysis. GraphPad Prism and Excel were used for statistical calculations. Significant differences are indicated using the standard Michelin Guide scale (*P* < 0.05, significant; *P* < 0.01, highly significant; *P* < 0.001, extremely significant).

## SUPPLEMENTARY FIGURES AND TABLES


