# Supplementary material for: Mitochondrial p32 is upregulated in Myc expressing brain cancers and mediates glutamine addiction
Source: Oncotarget. 2014 Dec 22;6(2):1157–70. doi: 10.18632/oncotarget.2708 (PMC4359224; doi:10.18632/oncotarget.2708)
Supplement: Supplementary file 1 [file oncotarget-06-1157-s001.pdf]

SUPPLEMENTARY FIGURES AND TABLES

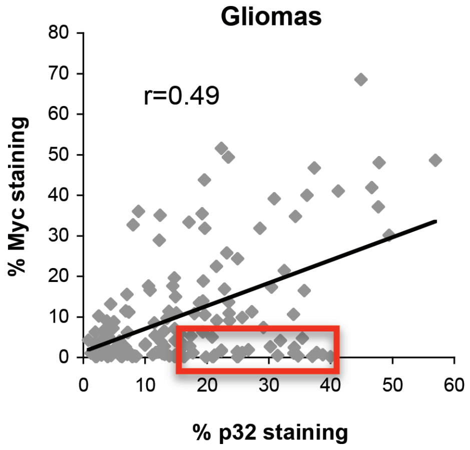

Supplementary Figure S1. Correlation between p32 and Myc expression in human gliomas. Sequential slides of a mixed glioma array were stained separately with polyclonal anti p32 and c-Myc antibodies. The % of p32 and Myc positive staining for each core was quantified using Aperio software. The indicated Pearson correlation coefficient ($r = 0.49$) was calculated using the entire data sets of Myc and p32 staining. The red box indicates samples expressing low Myc (<15% of staining) but moderate-high p32 (>15% of staining) and which have been excluded for analysis in Fig. 2B.

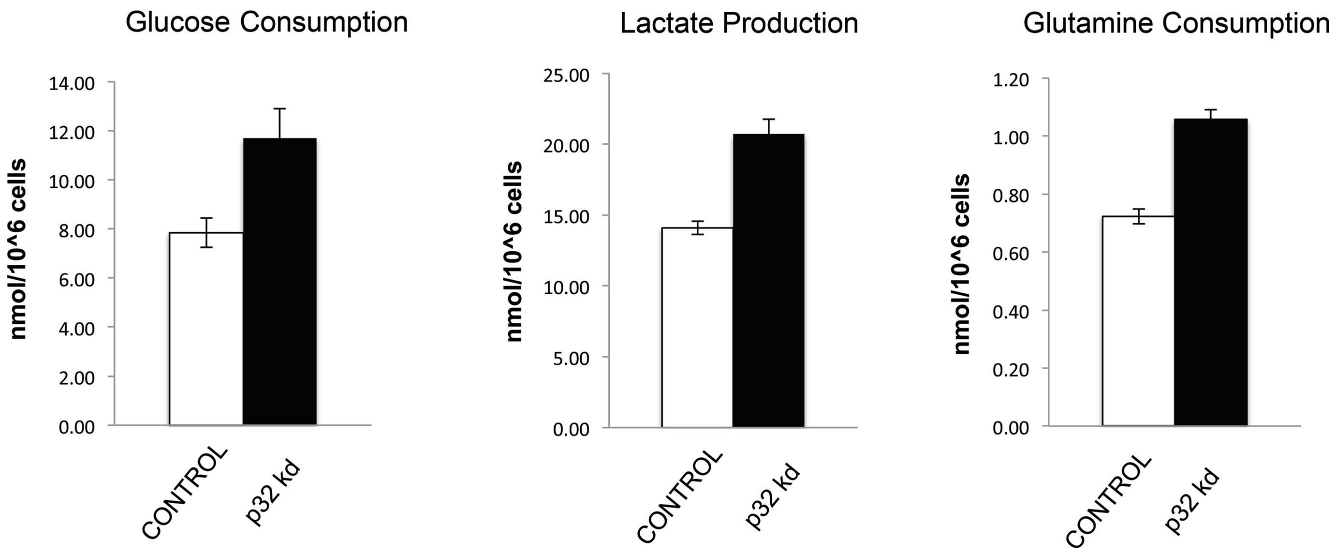

Supplementary Figure S2. Attenuation of p32 expression enhances glucose, and glutamine consumption and lactate production. SF188 cells were cultured in 25 mM glucose DMEM. 48 hours later, 500 µl of spent medium from each well was collected and metabolites glucose, lactate, and glutamine concentration was measured by the YSI 7100 MBS Multi Channel Biochemistry Analyzer. Data shown is the mean of three experiments ±SD.

Supplementary Table S1: List of primers used for quantitative real time PCR

	Forward Primer (5′- 3′)	Reverse Primer (5′- 3′)
P32	GAAGCGAAATTAGTGCGG	CCACGAAATTGGGAGTTGA
MYC	AGCGACTCTGAGGAGGA	AGACTCTGACCTTTTGCCA
CYCLIN D2	GCTGGCTAAGATCACCAA	CGGTACTGCTGCAGGCTAT
GLS1	CACTCAAATCTACAGGA	CTCCAGACTGCTTTTTAG
UBB	CCTGAGGGGTGGCTGTTAAT	GCTACCATGCAACGAAACCT
ACTIN	CATGTACGTTGCTATCCAG	CTCCTTAATGTCACGCACG

Supplementary Table S2: List of primers used for the Chromatin Immunoprecipitation (ChIP) experiment shown in Figure 4

	Forward Primer (5′- 3′)	Reverse Primer (5′- 3′)
E1	GCAAGAAAGCTCCGGAATCC	GCAGCATCGCGGAAACGAC
E2	CATTCCACGCAGCCCAAAAG	CCCTCTGTTGAACGCAAACCAC
Ex1	GCAGTCGTTTCCGCGATGCTG	GGTGCCGGCTGCAGGAGCTG
In1	GCCCATGGTCGTGGTTGAAAATAG	GTAGGAAGCTGAGCCTGATTCTGG
In3	CAGTTAATTGGCACATAGCTGAGGC	GTCAATACATGCTGCTGCTGGAAGG
